# Adenovirus Isolated From a Cat Is Related to Human Adenovirus 1

**DOI:** 10.3389/fmicb.2019.01430

**Published:** 2019-06-25

**Authors:** Joseph Ongrádi, Louise G. Chatlynne, Katalin Réka Tarcsai, Balázs Stercz, Béla Lakatos, Patricia Pring-Åkerblom, Donald Gooss, Károly Nagy, Dharam V. Ablashi

**Affiliations:** ^1^Department of Medical Microbiology, Semmelweis University, Budapest, Hungary; ^2^National Institute of Dermato-Venereology, Budapest, Hungary; ^3^Advanced Biotechnologies Inc., Columbia, MD, United States; ^4^Lakat-Vet BT, Budapest, Hungary; ^5^Department of Virology, Hannover Medical School, Hanover, Germany; ^6^Selbyville Animal Hospital, Selbyville, DE, United States

**Keywords:** feline adenovirus isolate, HAdV-1 related, hexon sequence, fiber sequence, cell host range, interspecies transmission, emerging pathogen

## Abstract

An adenovirus (AdV) has been isolated from the rectal swab of a domestic cat (*Felis catus*) and named feline adenovirus (FeAdV) isolate. It replicates and causes cytopathological effects in many human, feline, other mammalian cell lines that have both Coxsackie-adenovirus-receptor and integrins. Its antigens cross-react with anti-human adenovirus antibodies in immunofluorescence and immunocytochemistry assays. Electron microscopy revealed typical extracellular icosahedral particles and pseudo arrays inside cells. Sequence analysis of hexon and fiber genes indicates that this virus might belong to human adenovirus (HAdV) C species and might be a variant of type 1. In the fiber protein, three altered amino acids occur in the shaft; four altered residues are found in the knob region as compared to a European HAdV might be type 1 isolate (strain 1038, D11). One alteration affects amino acid 442 forming an RGS motif in an alanine rich region that might be an alternative way to bind integrins with subsequent internalization. Substitutions in the hexon sequence are silent. As compared to published HAdV sequences, the fiber is related to the original American prototype and recently described Taiwanese HAdV 1 isolates, but the hexon sequences are related to adenovirus isolates from France, Germany, Japan, and Taiwan. Serology carried out on FeAdV infected M426 cells indicates a prevalence of IgG in 80% of domestic cats in Delaware, United States. FeAdV isolate seems to be a recently recognized virus with possible pathogenic effects and, simultaneous human and feline infections are possible. Further molecular and biological characterization of this feline adenovirus isolate, as well as studies on both human and feline epidemiology and pathomechanisms, especially in endangered big cats, are warranted. FeAdV might have further practical advantages. Namely, it could be utilized in both human and feline AIDS research, developed into diagnostic tools, and gene therapy vectors in the near future.

## Introduction

Adenoviruses (AdV) are important, widespread [almost 100% of humans have circulating antibodies against the common human adenovirus (HAdV) types/see refs in Stercz et al., 2013], and occasionally fatal pathogens in humans, as well as wild and domestic animals. There are seven species (A through G) of Mastadenoviruses comprising a steadily increasing number of HAdV types. The HAdV Working Group has recognized 85 types recently^1^ [The HAdV Working Group (2019)]. Among these, species C is the most important since it can establish latent infection as episomes in immune cells and has a high fatality rate in immunocompromised patients. Reactivation of latent AdVs after bone marrow (BMT) or hematopoietic stem cell (HSCT) transplantation might elicit lethal complications or life-long sequelae (Lion, 2014). Early genes of AdVs have been shown to transactivate human immunodeficiency virus (HIV) (Kliewer et al., 1989), and consequently, promote AIDS progression. Before introducing the highly active antiretroviral treatment (HAART), approximately 20% of patients died of untreatable gastroenteritis (Hierholzer, 1992). Species C types can be excreted in massive amounts in the stool for months after initial infection (Matthes-Martin et al., 2013). Recombinant AdVs (rAdVs) and their immunomodulatory effects have been widely studied as gene transfer vehicles. Modified or recombinant AdV obtained from animal sources could be less immunogenic and provide long-term expression of therapeutic genes (Stercz et al., 2013). Clinical importance and anthropozoonosis of adenovirus infection in major animal groups have not been elucidated. Despite apparent medical and veterinary importance, the pathomechanism has received little attention. One of the obstacles is that no ideal animal model exists for the simultaneous establishment of the underlying diseases and adenovirus latency with subsequent reactivation. Most AdVs studied have a very narrow *in vivo* and *in vitro* host range, although crossing animal host or tissue culture species barrier has been described (Lion, 2014).

The cat (*Felis catus*) as an experimental model has become important since the discovery of the feline immunodeficiency virus (FIV) and feline AIDS in 1986 (Pedersen et al., 1987) as the only natural small animal model for human AIDS (Elder et al., 2010). Beside anti-retroviral chemotherapy and vaccine trials, cats can be used to study the pathomechanism of interaction between retrovirus and several heterologous microbes through the course of feline AIDS (Ongrádi et al., 2013). AdV studies in cats or *Felidae* are hindered by the lack of data on natural infection and epidemiology, as well as research and diagnostic tools (Gonin et al., 1995). Early studies by electron microscopy suggested the presence of an AdV like virus particles in a black panther suffering from inclusion body hepatitis (Gupta, 1978) and a domestic cat suffering from disseminated adenovirus infection, feline leukemia virus (FeLV) infection and hepatic failure (Kennedy, 1993), but neither virus has been isolated nor has aspects of infection been studied. In the feline AIDS model, first, we studied field cats for possible natural infection. In the middle of 1990’s we screened 470 European (from Hungary, Italy, Netherlands, and Scotland) cats for the presence of anti-AdV antibodies using HAdV-1 hexon antigen in a home-made ELISA, and found 9.8–20.3% seropositivity (Lakatos et al., 1996; Lakatos et al., 2000), in 162 American (CA) cats 26% positivity (Lakatos et al., 2000). Immunization by using purified HAdV-1 hexon protein induced a high titer of antibodies (>1:3200) in specific pathogen-free cats (Lakatos et al., 2000). In the second step, pharyngeal and rectal specimens from several seropositive domestic cats were screened for AdV DNA by nested PCR assay using consensus primers (Lakatos et al., 1997, 1999). The last step was to attempt isolation of a replication competent virus and its basic characterization. We managed to isolate an AdV from the PCR positive rectal specimen (Ongrádi, 1999). Virions were visualized by electron microscopy. The genes of hexon and fiber proteins, which determine AdV tropism and type specificity, were sequenced using species-specific and type-specific PCR. Gradually we have carried out biological studies. The range of permissive cells of human and animal origin was defined *in vitro*. Intracellular AdV antigens were shown by immunofluorescence (IFA) and immunocytochemistry (ICH) assays. Sequencing and biological probes suggest that the isolate is related to HAdV type 1. It is described as feline adenovirus (FeAdV) isolate. This isolate can also be numbered as FeAdV-1, as more isolates can be expected in the future.

## Materials and Methods

### Data Availability

Publicly available datasets were analyzed in this study. This data can be found here: http://www.ncbi.nlm.nih.gov/genbank/.

### *In vitro* Cultivation of the Feline Adenovirus Isolate

Pharyngeal and rectal swabs were taken from a cat seropositive and PCR positive for AdV (Lakatos et al., 1999), and immersed in 2 mL RPMI-1640 with 10% heat-inactivated fetal bovine serum (FBS) and 50 μg/mL gentamicin (Sigma, St. Louis, MO, United States) at 4°C. No adenovirus was being grown in the laboratory where and when the feline virus was isolated. After filtering through an 0.22 μm disposable filter (Millipore, Bedford, MA, United States), this inoculum was added to a culture of HeLa 90% confluent cells for 1 h at 37°C, and then the culture was adjusted to 5 mL with DMEM, 2% FBS. After 7–14 days detached cell suspensions were transferred to fresh flasks for three blind passages. One of the triplicate cultures developed cytopathic effect (CPE) after the third blind passage, and part of that culture’s supernatant was filtered through a 0.45 μm filter. These filtrates were used to infect fresh HeLa and VERO cultures resulting in the appearance of the same CPE. Next, several human and animal cells lines were tested for adenovirus infection using a low multiplicity of infection (moi) to follow the gradual development of cytopathic effect due to the spread of the virus in consecutive generations. Mammalian epithelial and fibroblast cultures were grown in DMEM, immune cells were maintained in RPMI-1640 with 10 mM HEPES, both medium completed with 10% FBS and 40 mikrog/ml gentamicin, while 2% or 7% FBS (Sigma, St. Louis, MO, United States), respectively, were added postinfection. Clarified supernatants of HeLa, CRFK, and M426 cells were used as virus stocks for subsequent infection of other cultured cells. For infection, 1 ml virus stock containing 2–4 × 10^4^ TCID_50_ (equals to 0.007–0.015 moi.) was used in a 25 cm^2^ tissue culture flask. CPE was scored up to 14 days (Tables 1, 2). Permissivity was assayed by immunofluorescence and immunocytochemistry assays (IFA and ICH, see below). Mock infected cells were adenovirus negative through the isolation process and permissivity studies tested by the same assays. Virus stocks were stored at -20 and -70°C.

**Table 1:** Replication of the feline adenovirus in different animal cell lines.

Species	Origin of cells	Cell line	Cytopathic effect	Viral antigen
Chinese hamster (*Cricetulus griseus*)	Ovarian epithelial	CHO-K1 (ECACC 85050302)	No CPE	IFA-, IHC-
Mouse (*Mus musculus*)	Fibroblast	3T3-L1 (ATCC CL-173)	No CPE	IFA-, IHC-
Rabbit (*Oryctolagus cuniculus*)	Kidney epithelial	RK-13 (ATCC CCL-37)	CPE+	IHC+
Cat (*Felis catus*)	Kidney epithelial	CRFK (ATCC CCL-94)	CPE+	IFA+, IHC+
Dog (*Canis familiaris*)	Kidney epithelial	MDCK (ATCC CCL-34)	CPE+	IHC+
Pig (*Sus scrofa*)	Kidney epithelial	PD-5 (ECACC 93120830)	CPE+	IFA+
	Kidney epithelial	PK-15 (ATCC CCL-33)	CPE+	IFA+
Rhesus macaque (*Macaca mulatta*)	Fibroblast (lung)	DBS-FRhL-2 (ATCC CL-160)	CPE+	IFA+
Green monkey (*Cercopithecus aethiops*)	Kidney epithelial	VERO (ATCC CCL-81)	CPE+	IFA+
	Kidney epithelial	COS7 (ATCC CRL-1651)	CPE+	IFA+, IHC+
Owl monkey (*Aotus trivirgatus*)	Kidney epithelial	OMK (ATCC CRL-1556)	CPE+	IFA+

*ATCC, American type culture collection; ECACC, European collection of cell cultures; CPE, cytopathic effect; IFA, immunofluorescent antibody; IHC, immunohistochemistry.*

**Table 2:** Replication of the feline adenovirus in different human (*Homo sapiens*) cell lines.

Type of cells	Origin of cells	Cell line	Cytopathic effect	Viral antigen
Fibroblasts	Lung fibroblast	M426 (ATCC PTA-5244)	CPE+	IFA+
	Primary human foreskin fibroblasts	–	CPE+	IFA+
Epithelial cells	Cervical cancer	HeLa (ATCC CCL-2)	CPE+	IFA+
	Embryonal kidney	293 (ATCC CRL-1573)	CPE+	IFA+
	Breast adenocarcinoma	MCF7 (ATCC HTB-22)	CPE+	IFA+
	Melanoma	MEWO (ATCC HTB-65)	^∗^CPE-/+	^∗^IFA-/+
Neural cells	Glioblastoma-astrocytoma	U87 (ATCC HTB-14)	CPE+	IFA+
	Glioblastoma	A-172 (ATCC CRL-1620)	CPE+	IFA+
Leukocytes	Human cord blood mononuclear cells (primary)	–	Degeneration, No CPE	IFA+
	Adult human peripheral blood lymphocytes (primary)	–	Degeneration, No CPE	IFA+
B-lymphocytes	Mature B-cell line	Bjab (DSMZ ACC 757)	No CPE	IFA+
	EBV transformed B-cell line	LCL- 8664 (ATCC CRL-1085)	No CPE	IFA+
	Human histiocyte lymphoma	U937 (ATCC CRL-1593.2)	No CPE	IFA+
T-lymphocytes	Acute lymphoblastic leukemia	MOLT-3 (ATCC CRL-1552)	No CPE	IFA+
	Acute lymphoblastic leukemia	HSB-2 (ATCC CCL-120.1)	No CPE	IFA+
	Lymphoblastic lymphoma	Sup-T1 (ATCC CRL-1942)	No CPE	IFA+
	Acute T-cell leukemia	E6.1 (ATCC TIB-152)	No CPE	IFA+

*ATCC, American type culture collection; ECACC, European collection of cell cultures; DSMZ, Deutsche Sammlung von Mikroorganismen und Zellkulturen GmbH/German collection of microorganisms and cell cultures; CPE, cytopathic effect; IFA, immunofluorescent antibody; IHC, immunohistochemistry. ^∗^MEWO, intracellular virus antigens and cytopathic effect developed from 7 days Postinfection.*

### Purification and Concentration of the Feline Adenovirus Isolate

HeLa cells were cultured in T75 tissue culture flasks until they reached 90% confluency. After infection with feline adenovirus, cells were cultured until cytopathic effect became visible as rounding of cells. Before the cells detached from the surface they were washed into the medium by pipetting, and then subsequently centrifuging at 1,000 *g* for 5 min. The supernatant was decanted, and cells were resuspended in the remaining supernatant. Viruses were released from the cells by three freeze/thaw cycles. Viruses were harvested by centrifugation at 3,000 *g* for 10 min then the supernatant was collected and clarified through a 0,45 μm filter. Feline adenovirus isolate was purified and concentrated with the purification filter (ViraBind^TM^ Adenovirus Purification Kit, Cell Biolabs, Inc., San Diego, CA, United States) subsequently eluted with the elution Buffer (25 mM TRIS, pH 7.5, 2,5 mM Mg_2_Cl, 1 M NaCl). For storage, 10% sterile glycerol was added. The final concentration of the virus was 1.35 × 10^7^ infectious unit/ml as titered on HEK 293 cells. Cells were infected with 10-fold serial dilution of feline adenovirus. At 48 h postinfection, before CPE becomes visible, intracellular hexon antigens were detected by immunocytochemistry (see below). Based on the average number of infected cells per microscopic field and the dilution factor, the viral titer was calculated as infectious unit/ml according to the following formula.

$$\text{Infectious unit/ml (ifu/ml)}=\frac{\left(\text{Average positive cells/field})\times (\text{number of fields/well})\times (\text{dilution factor}\right)}{0.1\text{ ml}}$$

The data were expressed as mean ± standard deviation (SD).

### Immunofluorescence Assays (IFA) to Detect Virus Antigens and Antiviral Antibodies

Infected and uninfected control cells of various cell lines (Tables 1, 2) were washed in phosphate buffered saline (PBS) and spotted on Teflon coated slides, air-dried, fixed in cold methanol-acetone and incubated with 10 μL of Adenovirus monoclonal antibody (Bartels VRK, Intracel, Issaquah, WA, United States), washed and stained with FITC conjugated anti-mouse IgG with and Evans blue counterstain as the secondary antibody. The cells were screened for specific immunofluorescence with a UV microscope. Human embryonic kidney 293 (HEK 293) cells infected with HAdV-2 and HAdV-7 were used as positive controls. Mock-infected cells and an irrelevant antibody to human herpesvirus 7 (RK4, Advanced Biotechnologies Inc., Columbia, MD, United States) served as negative controls. Furthermore, sera obtained from several male and female random domestic cats brought to the Selbyville Animal Hospital in Delaware United States were tested at a 1:20 dilution by IFA using the highly permissive M426 human cell line infected with FeAdV to assess the distribution of IgG antibody. Antibody-positive feline sera were titered.

### Immunocytochemistry Staining (ICH)

The presence of intracellular adenovirus hexon antigen in selected cell cultures was also visualized by immunoassay (Quick Titer Adenovirus Titer Immunoassay Kit, Cell Biolabs, Inc., San Diego, CA, United States). Cells at a concentration of 4 × 10^4^ cells/well were seeded in a 96-well tissue culture plate and, at 90% confluency, they were infected with the purified feline adenovirus at 5 moi. On day 3, when CPE was detected, the culture medium was decanted. Cells were fixed by methanol at -20°C for 20 min; subsequently washed with phosphate buffered saline (PBS) three times for 5 min each then blocked with 1% bovine serum albumin (BSA) in PBS for 1 h at room temperature on an orbital shaker. After removing the blocking solution, cells were incubated with the anti-hexon antibody for 1 h at room temperature on an orbital shaker. After washings (see above) cells were incubated with the horseradish peroxidase (HRP)-conjugated secondary antibody at room temperature for 1 h on an orbital shaker followed by washing five times. Wells were stained with freshly diluted 3,3′-diaminobenzidine (DAB) working solution for 10 min to visualize infected cells. Finally, wells were washed with PBS twice. Cells were examined in a light microscope. Infected cells were brown; the non-infected were not stained. The same negative and positive controls were used as in IFA studies.

### Electron Microscopy (EM)

After infection, when maximal CPE was achieved, CRFK, HeLa, PD-5, and M426 cells (4–5 days post infection) were centrifuged and fixed in glutaraldehyde-paraformaldehyde. Thin sections were stained. Supernatant samples were processed for negative staining. Both infected and uninfected samples were studied electron microscopically as described (Ongrádi et al., 2000).

### Polymerase Chain Reaction (PCR), Sequencing of the Hexon and Fiber Genes and Aligned With Published Sequences

Hexon specific PCR was carried out for subgenus and type determinations in Hanover, Germany in 1999. FeAdV was propagated several times in HeLa cultures. Viral DNA was prepared. The sample was tested for species specificity in PCR using a series of primers HsgA1 to HsgF2. DNA of serotypes belonging to the corresponding species were controls as described (Pring-Åkerblom et al., 1999). Since the feline sample showed positivity with species C primers, studies were extended to specific primers for HAdV-1, 2, and 5 (Pring-Åkerblom et al., 1997a). Next, the loop 1_4_ region and parts of the flanking conserved hexon gene regions were amplified with H1_4_/H1_4_2 primer pairs (Pring-Åkerblom et al., 1999). Primer pairs specific for HAdV-1 knob region were used to detect the fiber gene of the feline isolate as published (Pring-Åkerblom and Adrian, 1995a). PCR products of the hexon loop 1_4_ region were cloned into pUC18. Similarly, the complete hexon and fiber genes were sequenced using specific internal primers (Pring-Åkerblom and Adrian, 1993; Pring-Åkerblom et al., 1995, 1997b). DNA sequencing of an European HAdV-1 isolate [Netherlands, 1970, strain code 1038, genotype D11 (cited as D11)] (Adrian et al., 1990; Pring-Åkerblom et al., 1999) was carried out in the same way, and sequence alignment was done using DNASIS (Pharmacia, Uppsala, Sweden) as described (Pring-Åkerblom and Adrian, 1995b; Pring-Åkerblom et al., 1999). Recently, fiber and hexon sequences have been aligned with counterparts published in GenBank (Figures 10, 11) using Multiple Sequence Comparison by Log-Expectation [MUSCLE] (2018) with parameters provided in MUSCLE. The MEGA 7.0 (Molecular Evolutionary Genetic Analysis) software was used to generate a phylogenetic neighbor-joining tree applying the Neighbor-joining method using a Kimura-2-parameter model for calculating evolutionary distance, and 1,000 replicates for bootstrap analysis. Only those sequences could be matched that have corresponding regions in FeAdV, because in the case of other isolates, slightly longer or shorter fragments were available. The G + C ratio of available sequences was calculated by a formula (G + C)/(A + T + G + C) × 100, and compared to the homologous genes of known isolates.

## Results

### Cell Biology

**FIGURE 1:**
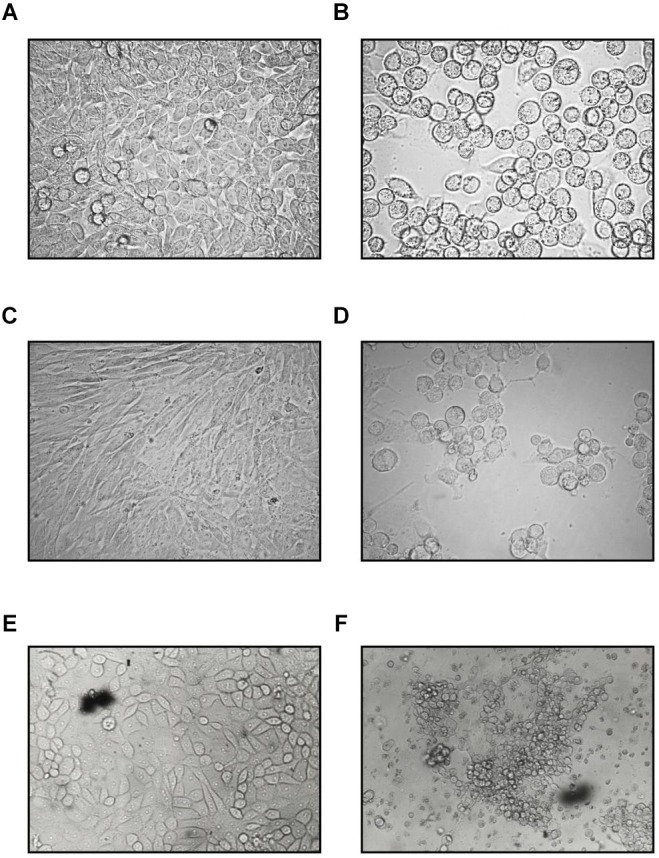
Cytopathic effect of the feline adenovirus isolate in permissive cells as compared to uninfected cells on day 5 postinfection (400×). Uninfected **(A)** and infected **(B)** HeLa cells, uninfected **(C)** and infected CRFK **(D)** cells, uninfected **(E)**, and infected **(F)** PD-5 cells. Infected cells form grape-like clusters.

Some “toxic” effect was seen at the edge of the HeLa monolayers for a few days post inoculation with the rectal specimen. During the third passage of this culture, the cytopathic effect (CPE) developed. The cells became refractile, rounded up, and formed grape-like clusters typical of species C type AdV. Transferring these cells or their cell-free supernatants (either filtered or unfiltered) to fresh HeLa cultures elicited the same CPE in 3–4 days (Figure 1). The virus titers from the supernatants of the HeLa cells peaked at 4 × 10^4^, 50% tissue culture infectious dose (TCID_50_) on day 5. This result shows that an infectious agent is obtained from the feces of the cat.

Meanwhile, another test showed that the infectious agent is an adenovirus. They have a very narrow host range. It was logical to establish the host range of the isolate, at least *in vitro*. Several human, feline and other mammalian cells lines proved to be permissive for the feline virus isolate, although there were some differences in timing and presentation of CPE (Tables 1, 2). On day 5, the virus titer produced by VERO cells reached its maximal level at 2 × 10^4^ TCID_50_. Human anti-AdV antibodies in immunofluorescent assays showed strong fluorescence in both nucleus and cytoplasm, as well as in the cytoplasmic membrane. The course of infection, peak titers, and distribution of intracellular virus antigens were similar in M426, HEK 293, OMK, rhesus monkey fibroblasts, CRFK and PD-5 and other mammalian cells to those found in HeLa and VERO cultures.

**FIGURE 2:**
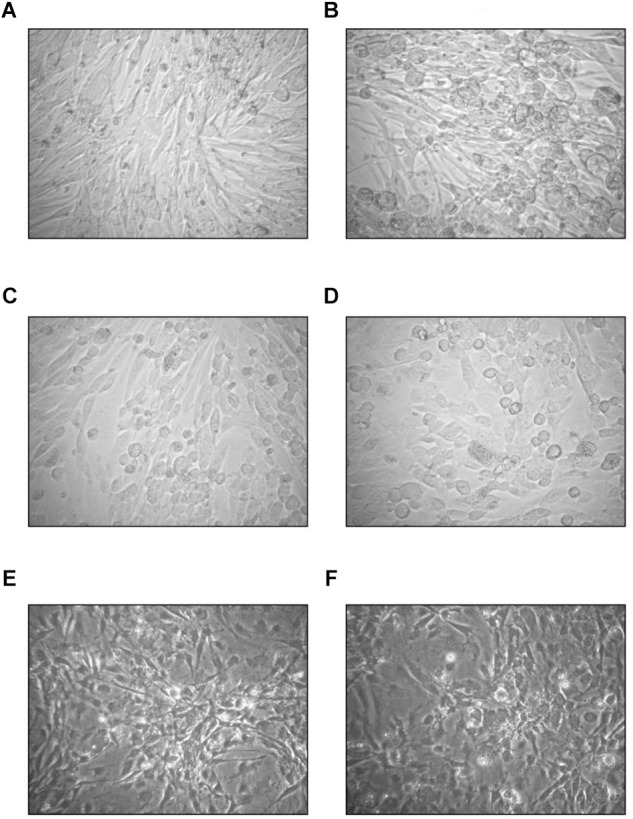
Effect of the feline adenovirus isolate in semi- and non-permissive cells as compared to uninfected cultures (400×). On day 7, uninfected **(A)** and infected **(B)** MEWO cells, on day 3, uninfected **(C)** and infected **(D)** CHO-K1 cells, on day 4 using phase contrast illumination, uninfected **(E)** and infected **(F)** 3T3-L1 cells.

**FIGURE 3:**
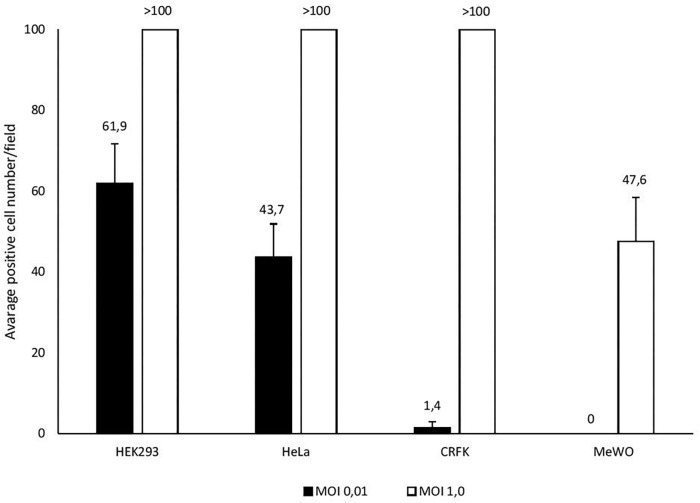
Different susceptibility of cell lines (HEK293, HeLa, CRFK, and MEWO) to feline adenovirus infection detected by immunocytochemistry. Cells were infected with different multiplicity of infection and positively stained cells were counted in a microscopic field 48 h post infection.

In U-87 cells, a neural cell line, CPE appeared on day 5 and reached 100% maximum on day 7, while in A-172 cells, another neural cell line, it became apparent on day 7 and reached maximal level at 75% in 10 days. In MEWO cells, the first sign of CPE was observed on day 7 only; it progressed very slowly as compared to the CPE seen on HeLa and CRFK cultures. Maximal CPE developed on day 10 (Figure 2). To compare adenovirus replication in HeLa, CRFK, and MEWO cells, 0.01 and 1.0 moi inocula were used to infect cultures, followed by virus titration during replication. FeAdV replication in CRFK cells depended on the input virus: 0.01 moi hardly resulted in virus replication, while 1.0 moi elicited FeAdV replication as high as in HeLa cultures. MEWO cells could be regarded as semi-permissive due to extremely slow adenovirus replication (Figure 3). No CPE were detected in CHO and 3T3-L1 cultures (Figure 2).

Human adult peripheral blood lymphocytes and cord blood lymphocytes did not exhibit CPE, but the degeneration of infected cultures was observed, and the IFA staining was restricted to the cytoplasm. No CPE and weak cytoplasmic fluorescence was detected in the human T lymphoid E6.1, HSB-2, MOLT-3, Sup-T1, B lymphoid Bjab and LCL and monocytic U-937 cultures, suggesting that these unstimulated cells may be non-permissive for FeAdV replication (Table 2).

### Immunofluorescence, Immunohistochemistry, and Electron Microscopy

**FIGURE 4:**
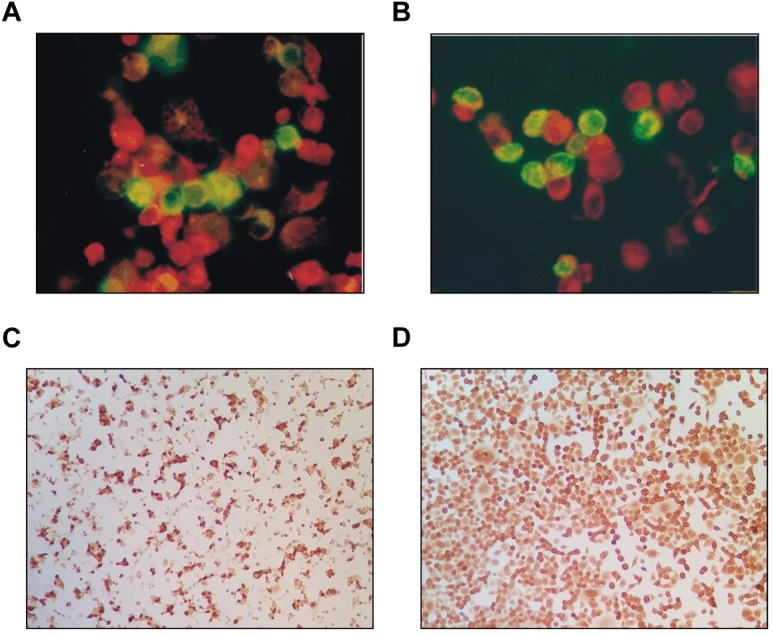
Adenovirus antigens detected in both the nucleus and cytoplasm of CRFK cat kidney cells **(A)** and HEK293 (human embryonic kidney) **(B)** by FITC-conjugated anti-human adenovirus antibody as compared to uninfected cells counterstained red (400×). The same antigens were detected in CRFK **(C)** and HeLa **(D)** cells by immunocytochemistry (dark cells).

Verification of the infectious agent occurred partially by classical antigen detection. Using anti-adenoviral antibodies, immunofluorescence and immunocytochemistry resulted in identical positive or negative results (Tables 1, 2). Adenovirus antigens were detected in all mammalian cells tested (Figure 4), except in CHO-K1 and 3T3-L1 cultures. Mock-infected cells remained negative. An irrelevant monoclonal antibody to human herpes virus 7 (RK4, 1:50 dilution) resulted in no fluorescence in cells with or without CPE.

**FIGURE 5:**
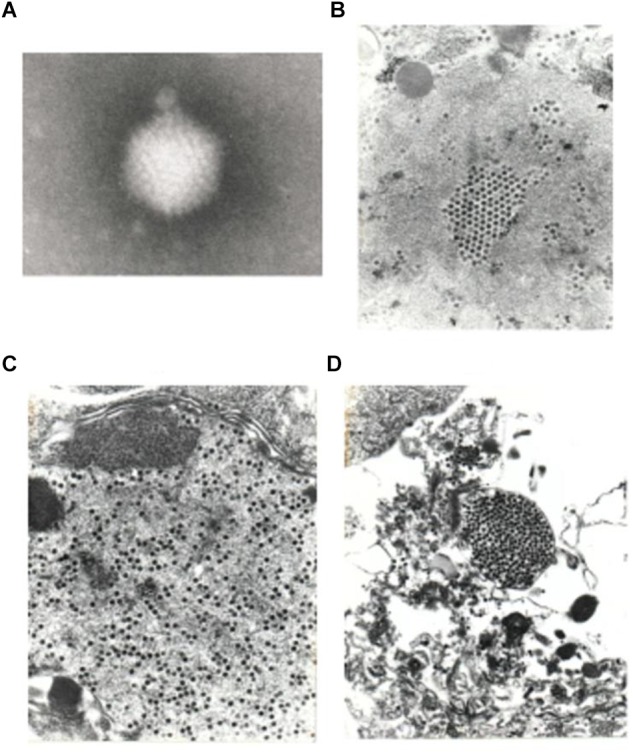
Electron micrograph of a negatively stained virus particle obtained from the supernatant of infected PD-5 culture **(A)**. The icosahedral shape and size (88 ± 4 nm) is characteristic for adenoviruses. Fibers could not be visualized (80,000× plus picture magnification). A pseudo array formed by adenoviruses in the nucleus of a PD-5 cell **(B)**, viruses scattered in the cytoplasm **(C,D)** (10,000× plus picture magnifications).

**FIGURE 6:**
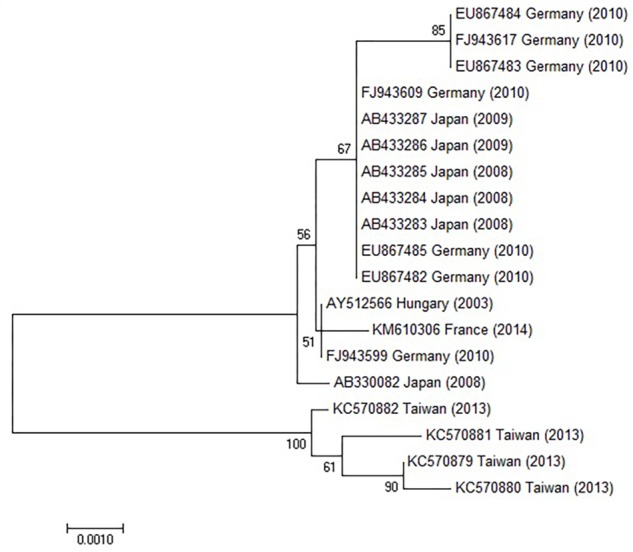
Phylogenetic tree constructed using HAdV-1 hexon genes retrieved from GenBank. The bar indicates the number of substitutions per site.

Supernatants of infected HeLa and M426 cells stored at -20°C for 3 years, and other supernatants of HeLa and CRFK cells stored at -20 or -70°C for 13 years resulted in the same level of infectivity as fresh virus stocks did, which demonstrates that the virus is very stable. Supernatants of infected PD-5 cells contained a large number of 88 ± 4 nm icosahedral particles resembling adenoviruses, but their fibers could not be visualized (Figure 5). The cytoplasm of infected PD-5, M426, HeLa, CRFK cells contained identical paracrystal arrays characteristic for adenoviruses (Figure 6). Electron microscopy showed that a single infectious agent was isolated and only this one infected and replicated in cell cultures.

Fifteen domestic feline sera from Delaware were tested by IFA on M426 cells infected with FeAdV isolate. Of these only three were completely negative. The remaining 12 showed staining varying from only nuclear to whole cell fluorescence; three sera had titers of 1:640 or greater. No IFA signal was seen when these sera were used to stain M426 cells that were not infected with FeAdV isolate. Any specific binding between feline antibodies and feline cellular antigens was avoided in IFA tests by using human M426 cells.

### Sequencing and Alignment of Hexon and Fiber Genes

**FIGURE 7:**
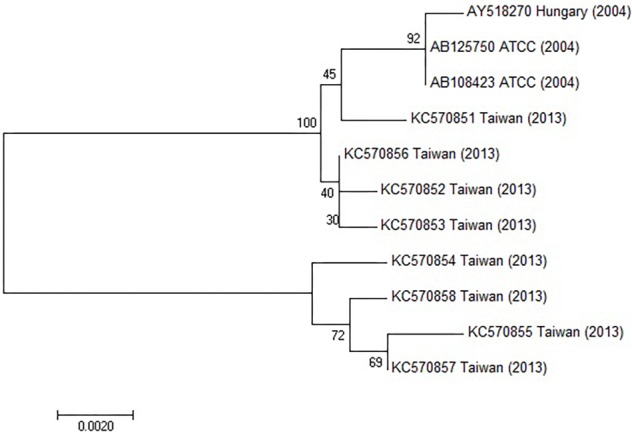
Phylogenetic tree constructed using HAdV fiber gene sequences retrieved from GenBank. The bar indicates the number of substitutions per site.

The hexon and fiber genes of the AdV isolate were sequenced due to their importance in adenovirus biology and classification. Species and type-specific PCR using species C and type HAdV-1 specific primers resulted in two amplimers one at bp 269 and the second at bp1049, respectively. Negative results were obtained using other primers. This indicates that the FeAdV isolate is closely related to HAdV-1. The hexon gene of the feline isolate was sequenced and compared to that of an HAdV-1 isolate [EMBL accession number X67709/26 (Pring-Åkerblom and Adrian, 1993)]. The mapped 2890 base pair region showed 99% identity with HAdV-1 (Supplementary Figure S1). Three nucleotide differences (bases 378, 1890, and 2196) in the feline sequence were in the last place of a triplet code and resulted in the same amino acids (aa) as are found in the human protein (Supplementary Figure S2). Sequence alignment with other published sequences shows that the hexon gene of the FeAdV isolate is most closely related to HAdV-1 (Figure 6) isolated in Marseille, France (Cassir et al., 2014), although its published sequence is 171 nt shorter at 3′ end and 341 nt at 5′ end. Two nucleotide differences were found. One was shown at nt 316 where C was replaced by T in the isolate from France, at nt 1,476 G was replaced by A in the same isolate. The homology of the comparable regions is 99.85%. The G + C content was higher (50.02%) in FeAdV isolate than in the isolate from Marseille (45.61%). None of the nucleotide alterations resulted in amino acid changes; leucine was coded at both sites. This phylogenetic tree based on hexon homology suggests that FeAdV isolate is related to several HAdV-1 isolates from Germany, Japan, and Taiwan (Figure 6). The sequence of the feline fiber gene was compared to the genotype of the D11 human counterpart (Supplementary Figure S3); it consisted of 1749 nucleotides that code for 582 amino acids (Supplementary Figure S4). There were a total of 12 differences in sequence found (Table 3), none in the tail (aa 1–44); three in the shaft (aa 45–401), all of which result in a change in amino acid, two with a change in charge; and nine in the knob region (aa 402–582). Of the alterations in the knob region, five were neutral for the amino acid sequence, and only one alteration resulted in a charge difference. One difference was in the conserved area of the knob; namely, amino acid 442 went from an arginine to a lysine. Differences resulting in an altered amino acid in the fiber protein affected the first nucleotide of the triplet code in four cases (base 220, 1015, 1240, and 1414), the middle nucleotide in two cases (base 596 and 1325), and the last nucleotide in one case (base 1581). Comparison of the fiber gene of FeAdV to that of other isolates shows that the feline isolate is most closely related to the first adenovirus isolated in 1953 (GenBank accession No AB125750, Adhikary et al., 2004a GenBank Accession No AB108423, Adhikary et al., 2004b, referring to adenoid71 by Rowe et al., 1953). Only one nucleotide alteration was found at position 981 in the shaft region without amino acid change; this is the last unit of that triplet code. Nucleotide homology between FeAdV isolate and adenoid 71 is 99.83%. The G + C content of the feline fiber is insignificantly higher (44.71%) than that of the isolate from Germany or Rowe’s isolate (44.28%). Interestingly enough, isolates from Taiwan are relatively closely related to FeAdV fiber gene (Figure 7).

## Discussion

Eighty percent of feline sera tested for antibodies were positive suggesting a very high prevalence of this strain of FeAdV in United States domestic cats. High percentage of seropositive sera using the FeAdV as antigen in comparison with 9.8–26% positivity using HAdV-1 as antigen (Lakatos et al., 1996, 2000) clearly shows that, the structure of antigens in the two AdVs is different, and the feline antibodies possess a higher avidity to the antigens of the feline AdV. This is in good correlation with the different amino acid sequence and consequent antigenicity between the human and feline AdV isolates. Following these pilot studies, more sera need to be tested especially those from cats showing some clinical manifestations. A limitation through the course of feline adenovirus serological studies is that human hexon antigen was used for testing adenovirus serology in European and American cats (Lakatos et al., 1996) by ELISA, but FeAdV antigen was used to test serology in the recent pilot study by IFA. Although the results unambiguously suggest important avidity differences, using both methods for testing all sera could be ideal in the future.

HeLa cells were used for isolation due to their high sensitivity for AdVs and, due to the lack of information on feline adenovirus cultures, reagents, diagnostic tools. The difference in the permissiveness for FeAdV of the various cell lines tested may be due to characteristics of the cells themselves. Identical CPE, IFA, and IHC positivity suggest that this feline adenovirus isolate can infect and replicate in several cell lines, that express both Coxsackie-adenovirus receptor (CAR, Howitt et al., 2003) and α_v_β_3_ or α_v_β_5_ integrins as coreceptors (Wickham et al., 1993). MDCK cells express CAR in the tight junctions, but FeAdV was able to infect them possibly by opening tight junctions (Sharma et al., 2012). CHO (Coyne and Bergelson, 2005) and 3T3-L1 cells (Orlicky and Schaack, 2001) cannot be infected by FeAdV, due to lack of CAR expression. The difference in the permissivity of CRFK cells using high inocula and low permissivity using small inocula might be an important finding in our study. Infection of CRFK cells with fowl adenovirus using relatively small inoculum (moi 1) resulted in binding of adenovirus particles through their CAR-dependent long fiber, but no virus replication was detected (Taharaguchi et al., 2012). CAR-dependent adenovirus should bind to integrins α_v_β_3_ or α_v_β_5_ for infection and replication on the surface of CRFK cells (Mahalingam et al., 2014). One can presume that in our experiments, a small amount of FeAdV inoculum either could not open cellular tight junctions for an autocatalytic availability of CAR molecules (Sharma et al., 2012) or could not activate integrin molecules from their inactive state (Mahalingam et al., 2014). Semi-permissivity of MEWO cells might be due to low expression of integrins that take part in AdV internalization (Menter et al., 1995), although these cells were successfully infected by HAdV type 5 vectors (Oka et al., 2002; Sarkar et al., 2008). In good correlation with our results, it is known that species C of HAdV has a low affinity for neural cells because the primary cell receptor for the virus, CAR expression is highly variable on neural cells (Skog et al., 2002).

The IFA and ICH tests detected FeAdV antigens in human leukocytes, B and T lymphocyte cultures suggesting that the feline adenovirus can infect these cells. On the contrary, CPE was not observed in the same immune cells meaning that, no significant virus replication took place in these cultures. The observed phenomenon is in good correlation with earlier publications, namely that, the serotypes of species C are known to establish lifelong persistence in lymphoid tissues and, cellular stimuli are required to reactivate viruses, and elicit virus replication (Lauer et al., 2004).

**Table 3:** Difference in base sequence and resulting amino acid sequence of feline adenovirus fiber protein as compared to analogous human adenovirus (HAdV) type 1 nucleotides and proteins.

Base #	Feline base	D11 human base	ATCC AB125750 base	Amino acid #	Location	Feline amino acid (charge)	D11 human amino acid (charge)	ATCC 125750 amino acid
Shaft portion (amino acids 45–401)								
220	A	G	A	74	*2. pseudorepeat*	Lysine (+)	Glutamic acid (-)	*Lysine (+)*
596	A	G	A	199	*9. pseudorepeat*	Asparagine	Serine	*Asparagine*
981 (1047^∗^)	A	A	G	327	*17. pseudorepeat*	*Lysine (+)*	*Lysine (+)*	*Lysine (+)*
1015	C	A	C	339	*18. pseudorepeat*	Histidine (+)	Asparagine	*Histidine (+)*
Knob portion (amino acids 402–582)								
1240	C	T	C	414	AB loop	Histidine (+)	Tyrosine	*Histidine (+)*
*1248*	*C*	*T*	*C*	*428*	*BC loop*	*Cysteine*	*Cysteine*	*Cysteine*
1325	G	A	G	442	CD loop	Arginine (+)	Lysine (+)	*Arginine (+)*
*1341*	*T*	*C*	*T*	*447*	*CD loop*	*Proline*	*Proline*	*Proline*
*1395*	*A*	*G*	*A*	*465*	*DE loop*	*Glycine*	*Glycine*	*Glycine*
1414	A	G	A	472	DE loop	Serine	Glycine	*Serine*
1581	A	T	A	527	GH loop	Glutamic acid (-)	Aspartic acid (-)	*Glutamic acid (*-*)*
*1659*	*C*	*A*	*A*	*553*	*HI loop*	*Serine*	*Serine*	*Serine*
*1743*	*G*	*A*	*A*	*581*	*Carboxy terminal*	*Glutamine*	*Glutamine*	*Glutamine*

*^∗^Nucleotide number of AB125750 published in ATCC. Silent amino acid alterations as compared to the feline amino acids are shown in italic.*

Surprisingly and uniquely, FeAdV replicated well in several mammalian cell lines demonstrating a wide cell tropism. As the alterations in the hexon gene did not result in any change in amino acid sequence, it is unlikely that this protein showed any functional change. For explaining this unusual phenomenon, it is speculated that the amino acid differences from the human sequence in the fiber might account for the expanded tropism. The fiber is the major determinant of tropism, the globular knob region of the fiber polypeptide attaches to CAR (Pring-Åkerblom and Adrian, 1995a,b; Eiz and Pring-Åkerblom, 1997; Roelvink et al., 1999). Each knob molecule contains b-strands (A to J) connected by prominent loops. Several conserved amino acid residues on the AB loop, B b-strand, CD-, DE-, FG-, and HI loops are required for binding to CAR (Roelvink et al., 1999), although contact residues are not well conserved (Howitt et al., 2003). S408 and Y477 crucial binding residues are found in both HAdV-2 (Howitt et al., 2003) and our HAdV-1 D11 and FeAdV isolates. Although four amino acid differences from the human isolate affected the AB-, CD-, DE-, and GH loops (Table 2), only the change of amino acid 442 from lysine to arginine is likely to have any functional significance that might result in a change of tropism or pathogenicity (Pring-Åkerblom and Adrian, 1994, 1995b; Eiz and Pring-Åkerblom, 1997), because it is the only alteration in the conserved CAR binding area of the CD loop (Figure 7). Altered arginine 442 and glutamic acid 527 is identical to species E HAdV-4 having an arginine in the same position (Pring-Åkerblom and Adrian, 1995a,b; Pring-Åkerblom et al., 1998). Binding FeAdV to porcine cells line raises the possibility that its fiber knob possesses structures similar to the porcine (P) AdV-4 and PAdV-3 (Nagy et al., 2002). Among the CAR binding conserved sequences common in HAdV-1 and FeAdV, only four amino acids in the AB loop, two in the FG loop of PAdV-4 (Kleiboeker, 1995), three amino acids in PAdV-3 (Reddy et al., 1995), two amino acids of PAdV-5 AB or FG loops (Nagy et al., 2002) are identical. None of the altered amino acids of FeAdV fiber knob are found in any of the PAdV. These data exclude a direct relationship between FeAdV and PAdV. The structure of feline CAR is not yet known. CAR is highly conserved; the domain of human and porcine CAR that is known to interact with the fiber shows 93.7% homology (Griesche et al., 2008).

No differences in the amino acid structure were detected through alignment of FeAdV fiber with adenoid 71 virus or alignment of FeAdV hexon with the most related isolate from France. This suggests that FeAdV isolate might be relatively distant from the European HAdV-1 isolate D11, but could be phylogenetically nearer to American adenoid 71 (Rowe et al., 1953) and Asian isolates. Detection of FeAdV in a human sample in Japan also suggests this relationship (Phan et al., 2006). Higher G + C content in the hexon gene of FeAdV isolate than in the isolate from Marseille suggests that FeAdV hexon gene possesses increased genomic stability, although drawing conclusions from the limited span of the genome seems to be very early.

Two of the alterations in the fiber shaft resulted in gaining two excess basic amino acids in the 2nd and 8th pseudo repeat 15-amino acid motifs. It is presumed that this might cause a functional change allowing more flexibility of the rod-like shaft, thus allowing it to adapt to a wider variety in cell types as it has been known with other C types (Lion, 2014). Particle binding to CAR is followed by adjusting the arginine-glycine-aspartic acid (RGD) motif of the penton base to cell membrane integrins for subsequent internalization (Fechner et al., 1999). In recombinant AdV vectors, inserting an RGD motif into the HI loop results in CAR-independent cellular entry via integrins (Madisch et al., 2007). Insertion of RGD into the feline parvovirus also extends its tropism to human tumor cells (Maxwell et al., 2001). The fiber knob of PAdV-4 contains an RGD motif beginning at residue 361 and surrounded by upstream sequences rich in alanine and glutamic acid (Kleiboeker, 1995). This structure resembles the penton base of species C HAdV, in which the RGD motif with downstream alanine and glutamic acid-rich sequences mediate integrin binding (Fechner et al., 1999). In the fiber knob of FeAdV the altered amino acid 442 forms an arginine-glycine-serine (RGS) motif at the beginning of the CD loop which is surrounded by alanine residues at positions 438, 440, 446, and 445 and a glutamic acid at 463. Furthermore, two residues downstream the RGS motif in FeAdV and the RGD motif in PAdV-4 are identical: leucine-alanine (LA). These speculations raise the possibility that the feline RGSLA moiety might mediate an alternative virus internalization into cells with low integrin expression, as this could be the way of infection of MEWO cells.

Isolation of FeAdV suggests that an AdV can infect cats. A concern is whether this isolate is a cross-contaminant or really obtained from an animal in which virus replication occurred. The history of the cat yielded the FeAdV isolate suggests; it is not a contaminant. Cited as: “From a 2-year-old domestic cat kept as a single pet in isolation, pharyngeal and rectal swab samples were taken twice at a 12 months interval. At the beginning of the examinations, the cat suffered from transient hepatic failure. Subsequently, the animal was repeatedly examined by a group specific indirect ELISA test, and found highly seropositive for adenovirus hexon antigens throughout 18 months. … The first rectal and the rectal and pharyngeal samples taken 1 year later gave amplification bands of identical size with the positive control… The positive PCR results are suggestive of persistent infection and shedding of adenovirus in the examined animal… The DNA sequence of the three PCR products (GenBank Accession number AF172246 are identical)” (Lakatos et al., 1999). The first rectal sample was used for virus isolation. The 301 bp fragment of the hexon gene product obtained directly from the feces is identical to the sequence amplified from the virus isolate (GenBank Accession number AY512566) (Supplementary Figure S1).

The genome of FeAdV isolate spanning between the fiber and hexon genes and the first half of the genome has not been characterized; theoretically, they could carry several mutations, or be recombinants, as novel HAdV types have shown such structures (refs in Lion, 2014). Non-human related AdV types also might establish an opportunistic infection in immunocompromised cats, as sequencing of the small fragments of hexon and polymerase genes in an archived sample (Kennedy, 1993) has recently shown (Lakatos et al., 2017), but whole genome sequencing has not been done. Serological studies and viral DNA detection, isolation of a replication competent adenovirus from a cat and, its basic characterization are a milestone in the series of feline adenovirus studies. Lack of the whole genome sequencing can be seen as a limitation of both adenovirus studies. As a next milestone, complete sequence analysis could yield more information on the origin of feline adenoviruses.

Interspecies transmission and recombination of adenoviruses are not a curiosity, e.g., it has been documented in the case of HAdV-4 (Dehghan et al., 2013), for other cases in species Human mastadenovirus C see Virus Taxonomy 2018b Release (2018). The broad tissue permissivity and the few differences between the feline and human viral polypeptides also strongly suggests an anthropozoonosis from human to cat or vice versa. HAdV-1 is widespread all over the world. Consequently one of the possibilities is that the FeAdV isolate is a natural variant of HAdV-1 adapted to cats. A case report from Japan found the same AdV cluster in the fecal specimen collected from a 1-year-old girl with acute gastroenteritis. They shared 100 and 97% identities at the amino acid levels of hexon and fiber genes, respectively, with corresponding FeAdV genes. The virus was designated as 6277JP, amino acid sequence was submitted to DDRJ DNA/GenBank database (accession number DQ336392) (Phan et al., 2006). A very broad phylogenetic tree containing several human, mammalian and avian adenovirus “subgenus” and species was constructed. Feline adenovirus, HAdV type 1/Adenoid 71 (Prototype), HAdV type 1/6277JP clustered. This publication was the first to show a close relationship between FeAdV and HAdV-1 (Phan et al., 2006).

Furthermore, these authors conclude that their case is proof of the interspecies transmission of HAdV-1 variant as FeAdV isolate, from a cat to the child. Lately, one isolate from 468 upper respiratory tract specimens in Brazil has shown 100% hexon gene sequence homology to the counterpart of FeAdV (Luiz et al., 2010). Both publications suggest that FeAdV is prevalent all over the world and, can infect humans, but presumably, rarely does. In a recent publication, phylogenetic analysis limited to hexon and fiber sequences has shown that the most related isolate to FeAdV was found in an intensive care unit in Marseille, France. Bronchoalveolar lavage of patients with highly immunocompromised state following lung transplantation and mechanical ventilation showed PCR positivity. Presumably, a nurse taking care of patients without wearing a mask while exhibiting respiratory signs and symptoms might have been the source of the outbreak. As no other cases of HAdVs were reported in Marseille public hospitals and among relatives of health care workers (Cassir et al., 2014), it raises the possibility of zoonotic infection from the cat of the nurse. This aspect has not been explored in the above-mentioned epidemiological survey, but it will be a must in future cases!

Detection of HAdV-1 hexon PCR positivity must be followed by sequencing to distinguish HAdV-1 and FeAdV (Jothikumar et al., 2005). Sequencing the fiber-specific amplicon also could distinguish between human and feline AdVs as recommended for other AdVs (Lion, 2014). These methods could yield exact data on the global epidemiology of FeAdV. The *in vivo* effects of the feline isolate is not known for humans, cats or *Felidae* especially, endangered big cats; these ought to be explored. Immunocompromised hosts might be at a higher risk, e.g., simultaneous FeLV (Kennedy, 1993) or FIV (Lakatos et al., 2000) infection. FeAdV has exceptional value in AIDS research: it can be utilized in both human studies and the *in vivo* feline AIDS model As speculated, the flexibility of the fiber molecule predisposes FeAdV isolate for further genetic manipulations as a gene therapy vector, especially with oncolytic potential. Depending on availability of funds, whole genome sequencing of the feline isolate is planned for such purposes.

Based on published hexon and fiber sequences (Pring-Åkerblom and Ongrádi, 1980a,b) and previous publications listed above, FeAdV isolate was included in the 14th International Committee for Virus Taxonomy Database (ICTVdb) as 00.001. Adenoviridae, 00.001.0.01.010.00.101.901 HAdV 1, feline isolate HAdV-1 approved by the Adenoviridae Study Group (Fauquet et al., 2000). The archived copy is available on the internet. Criteria of the novel taxonomy have been changed since no isolates have been included in the taxonomy^2^ [Virus Taxonomy 2018b Release (2018)]. The feline adenovirus (Taxon identifier 114408) is listed in UniProt (2018) (UniProt accessed November 13, 2018). FeAdV isolate has been deposited in the American Type Culture Collection (ATCC) (AcqID-00755).

All previous and recent data taken together support the idea that cats can be infected by adenoviruses, viruses replicate in their cells, viruses are shed, an adequate immune response is mounted. We are aware of the fact that a single isolation cannot enlight the pathomechanisms of adenovirus replication in cats. Furthermore, limited information on the genetic and polypeptide structures of the single isolate cannot explain the molecular mechanism of its presumed interspecies transmission. Our considerations at the molecular level outlined here should draw attention to these particular aspects to analyze further isolates.

## Author Contributions

JO and DA designed the research. LC, KT, DA, PP-Å, BS, and KN conducted the research. BL and DG provided feline specimens. All authors listed had analyzed the data, made a substantial intellectual contribution to this work, and approved the manuscript for publication.

## Conflict of Interest Statement

DA and LC were employed by Advanced Biotechnologies Inc. (Columbia, MD, United States). The remaining authors declare that the research was conducted in the absence of any commercial or financial relationships that could be construed as a potential conflict of interest.

## Footnotes

1 http://hadvwg.gmu.edu

2 http://ictvonline.org
