# Supplementary material for: Adenovirus Isolated From a Cat Is Related to Human Adenovirus 1
Source: Front Microbiol. 2019 Jun 25;10:1430. doi: 10.3389/fmicb.2019.01430 (PMC6603132; doi:10.3389/fmicb.2019.01430)
Supplement: Supplementary file 1 [file Data_Sheet_1.PDF]

Supplementary Figure 1. Sequence alignment of the complete hexon genes of HAdV-1 (D11) and FeAdV.

```
HAdV-1    1   GCTACCCCTTCGATGATGCCGCAGTGGTCTTACATGCACATCTCGGGCCA     50
              ||||||||||||||||||||||||||||||||||||||||||||||||||
FeAdV     1   GCTACCCCTTCGATGATGCCGCAGTGGTCTTACATGCACATCTCGGGCCA     50

         51   GGACGCCTCGGAGTACCTGAGCCCCGGGCTGGTGCAGTTCGCCCGCGCCA    100
              ||||||||||||||||||||||||||||||||||||||||||||||||||
         51   GGACGCCTCGGAGTACCTGAGCCCCGGGCTGGTGCAGTTCGCCCGCGCCA    100

        101   CCGAGACGTACTTCAGCCTGAATAACAAGTTTAGAAACCCCACGGTGGCG    150
              ||||||||||||||||||||||||||||||||||||||||||||||||||
        101   CCGAGACGTACTTCAGCCTGAATAACAAGTTTAGAAACCCCACGGTGGCG    150

        151   CCTACGCACGACGTGACCACAGACCGGTCTCAGCGTTTGACGCTGCGGTT    200
              ||||||||||||||||||||||||||||||||||||||||||||||||||
        151   CCTACGCACGACGTGACCACAGACCGGTCTCAGCGTTTGACGCTGCGGTT    200

        201   TATCCCCGTGGACCGCGAGGATACCGCATACTCGTACAAGGCGCGGTTTA    250
              ||||||||||||||||||||||||||||||||||||||||||||||||||
        201   TATCCCCGTGGACCGCGAGGATACCGCATACTCGTACAAGGCGCGGTTTA    250

        251   CCCTGGCTGTGGGTGACAACCGTGTGCTTGACATGGCTTCCACATACTTT    300
              ||||||||||||||||||||||||||||||||||||||||||||||||||
        251   CCCTGGCTGTGGGTGACAACCGTGTGCTTGACATGGCTTCCACATACTTT    300

        301   GACATTCGCGGCGTGCTGGACCGGGGCCCCACTTTTAAGCCCTACTCCGG    350
              ||||||||||||||||||||||||||||||||||||||||||||||||||
        301   GACATTCGCGGCGTGCTGGACCGGGGCCCCACTTTTAAGCCCTACTCCGG    350

        351   CACTGCCTACAACGCTCTAGCCCCCAAGGGCGCTCCCAATTCCTGCGAGT    400
              ||||||||||||||||||||||||||| ||||||||||||||||||||||
        351   CACTGCCTACAACGCTCTAGCCCCCAAAGGCGCTCCCAATTCCTGCGAGT    400

        401   GGGAACAAGAAGAACCAACTCAGGAAATGGCTGAAGAACTTGAAGATGAG    450
              ||||||||||||||||||||||||||||||||||||||||||||||||||
        401   GGGAACAAGAAGAACCAACTCAGGAAATGGCTGAAGAACTTGAAGATGAG    450

        451   GAGGAGGCAGAGGAGGAGGAGGCAGAGGAGGAGGCAGAAGCACCACAAGC    500
              ||||||||||||||||||||||||||||||||||||||||||||||||||
        451   GAGGAGGCAGAGGAGGAGGAGGCAGAGGAGGAGGCAGAAGCACCACAAGC    500

        501   TGATCAGAAGGTTAAGAAGACTCATGTATATGCTCAGGCTCCTTTGGCAG    550
              ||||||||||||||||||||||||||||||||||||||||||||||||||
        501   TGATCAGAAGGTTAAGAAGACTCATGTATATGCTCAGGCTCCTTTGGCAG    550

        551   GGGAAAAAATTACCGCCAATGGCTTACAAATAGTTTCTGATACCCAAACT    600
              ||||||||||||||||||||||||||||||||||||||||||||||||||
        551   GGGAAAAAATTACCGCCAATGGCTTACAAATAGTTTCTGATACCCAAACT    600

        601   GAAGGCAATCCAGTTTTTGCCGATCCCACTTATCAACCTGAACCTCAGGT    650
              ||||||||||||||||||||||||||||||||||||||||||||||||||
        601   GAAGGCAATCCAGTTTTTGCCGATCCCACTTATCAACCTGAACCTCAGGT    650
```

```
 651  TGGAGAATCTCAGTGGAATGAAGCTGAAGCAACTGCATCTGGAGGCAGAG   700
      ||||||||||||||||||||||||||||||||||||||||||||||||||
 651  TGGAGAATCTCAGTGGAATGAAGCTGAAGCAACTGCATCTGGAGGCAGAG   700

 701  TACTAAAAAAGACTACTCCCATGAAACCATGCTACGGATCGTATGCCAGA   750
      ||||||||||||||||||||||||||||||||||||||||||||||||||
 701  TACTAAAAAAGACTACTCCCATGAAACCATGCTACGGATCGTATGCCAGA   750

 751  CCTACAAATAAAAATGGGGGTCAAGGTATACTAGTAGCAAACAACCAAGG   800
      ||||||||||||||||||||||||||||||||||||||||||||||||||
 751  CCTACAAATAAAAATGGGGGTCAAGGTATACTAGTAGCAAACAACCAAGG   800

 801  TGCTCTAGAGTCTAAAGTTGAAATGCAGTTTTTTGCCCCCTCTGGCACTG   850
      ||||||||||||||||||||||||||||||||||||||||||||||||||
 801  TGCTCTAGAGTCTAAAGTTGAAATGCAGTTTTTTGCCCCCTCTGGCACTG   850

 851  CCATGAATGAAAGAAATGCTGTTCAGCCAAGTATTGTTTTGTACAGTGAG   900
      ||||||||||||||||||||||||||||||||||||||||||||||||||
 851  CCATGAATGAAAGAAATGCTGTTCAGCCAAGTATTGTTTTGTACAGTGAG   900

 901  GATGTTAATATGGAAACTCCTGATACTCACATTTCATACAAACCAAGCAA   950
      ||||||||||||||||||||||||||||||||||||||||||||||||||
 901  GATGTTAATATGGAAACTCCTGATACTCACATTTCATACAAACCAAGCAA   950

 951  AACTGATGAAAACTCTAAGGCTATGTTGGGTCAACAAGCAATGCCAAACA  1000
      ||||||||||||||||||||||||||||||||||||||||||||||||||
 951  AACTGATGAAAACTCTAAGGCTATGTTGGGTCAACAAGCAATGCCAAACA  1000

1001  GACCCAATTACATCGCTTTTAGGGACAATTTTATTGGCCTTATGTATTAC  1050
      ||||||||||||||||||||||||||||||||||||||||||||||||||
1001  GACCCAATTACATCGCTTTTAGGGACAATTTTATTGGCCTTATGTATTAC  1050

1051  AACAGCACTGGTAACATGGGTGTCCTTGCTGGACAAGCATCACAGCTAAA  1100
      ||||||||||||||||||||||||||||||||||||||||||||||||||
1051  AACAGCACTGGTAACATGGGTGTCCTTGCTGGACAAGCATCACAGCTAAA  1100

1101  TGCCGTGGTAGACTTGCAGGACAGAAACACAGAGCTGTCATATCAACTTT  1150
      ||||||||||||||||||||||||||||||||||||||||||||||||||
1101  TGCCGTGGTAGACTTGCAGGACAGAAACACAGAGCTGTCATATCAACTTT  1150

1151  TGCTTGATTCTATTGGCGATAGAACCAGATACTTTTCCATGTGGAATCAG  1200
      ||||||||||||||||||||||||||||||||||||||||||||||||||
1151  TGCTTGATTCTATTGGCGATAGAACCAGATACTTTTCCATGTGGAATCAG  1200

1201  GCTGTAGACAGCTATGATCCAGATGTCAGAATCATTGAAAATCATGGAAC  1250
      ||||||||||||||||||||||||||||||||||||||||||||||||||
1201  GCTGTAGACAGCTATGATCCAGATGTCAGAATCATTGAAAATCATGGAAC  1250

1251  TGAGGATGAGTTGCCAAATTACTGCTTCCCACTTGGCGGTATAGGGGTAA  1300
      ||||||||||||||||||||||||||||||||||||||||||||||||||
1251  TGAGGATGAGTTGCCAAATTACTGCTTCCCACTTGGCGGTATAGGGGTAA  1300

1301  CTGACACCTACCAAGGTATAAAATCAAACGGAAACGGTAATCCTCAAAAC  1350
      ||||||||||||||||||||||||||||||||||||||||||||||||||
1301  CTGACACCTACCAAGGTATAAAATCAAACGGAAACGGTAATCCTCAAAAC  1350
```

```
1351  TGGACCAAAAATGACGATTTTGCGGCACGTAATGAAATAGGTGTGGGAAA  1400
      ||||||||||||||||||||||||||||||||||||||||||||||||||
1351  TGGACCAAAAATGACGATTTTGCGGCACGTAATGAAATAGGTGTGGGAAA  1400

1401  CAACTTTGCCCTGGAGATTAACCTTAATGCCAACCTATGGAGAAATTTCC  1450
      ||||||||||||||||||||||||||||||||||||||||||||||||||
1401  CAACTTTGCCCTGGAGATTAACCTTAATGCCAACCTATGGAGAAATTTCC  1450

1451  TCTACTCCAACATTGCACTGTACCTGCCTGACAAGCTAAAATACACTCCT  1500
      ||||||||||||||||||||||||||||||||||||||||||||||||||
1451  TCTACTCCAACATTGCACTGTACCTGCCTGACAAGCTAAAATACACTCCT  1500

1501  ACAAATGTGGAAATATCTCCCAACCCTAATTCATACGATTATATGAACAA  1550
      ||||||||||||||||||||||||||||||||||||||||||||||||||
1501  ACAAATGTGGAAATATCTCCCAACCCTAATTCATACGATTATATGAACAA  1550

1551  GCGAGTGGTGGCTCCCGGGTTGGTGGATTGCTACATTAACCTTGGAGCGC  1600
      ||||||||||||||||||||||||||||||||||||||||||||||||||
1551  GCGAGTGGTGGCTCCCGGGTTGGTGGATTGCTACATTAACCTTGGAGCGC  1600

1601  GTTGGTCATTGGACTACATGGACAACGTCAACCCCTTTAACCATCACCGC  1650
      ||||||||||||||||||||||||||||||||||||||||||||||||||
1601  GTTGGTCATTGGACTACATGGACAACGTCAACCCCTTTAACCATCACCGC  1650

1651  AATGCGGGCCTACGCTACCGCTCCATGTTGCTGGGCAACGGTCGCTACGT  1700
      ||||||||||||||||||||||||||||||||||||||||||||||||||
1651  AATGCGGGCCTACGCTACCGCTCCATGTTGCTGGGCAACGGTCGCTACGT  1700

1701  GCCCTTTCACATCCAGGTTCCTCAGAAGTTTTTTGCCATTAAGAACCTCC  1750
      ||||||||||||||||||||||||||||||||||||||||||||||||||
1701  GCCCTTTCACATCCAGGTTCCTCAGAAGTTTTTTGCCATTAAGAACCTCC  1750

1751  TACTCTTGCCGGGCTCATACACCTACGAGTGGAACTTCAGGAAAGATGTT  1800
      ||||||||||||||||||||||||||||||||||||||||||||||||||
1751  TACTCTTGCCGGGCTCATACACCTACGAGTGGAACTTCAGGAAAGATGTT  1800

1801  AACATGGTCCTGCAAAGCTCCCTAGGAAACGACCTAAGAGTTGACGGAGC  1850
      ||||||||||||||||||||||||||||||||||||||||||||||||||
1801  AACATGGTCCTGCAAAGCTCCCTAGGAAACGACCTAAGAGTTGACGGAGC  1850

1851  CAGCATTAAGTTTGACAGCATTTGCCTCTACGCCACCTTYTTTCCGATGG  1900
      ||||||||||||||||||||||||||||||||||||||| ||||||||||
1851  CAGCATTAAGTTTGACAGCATTTGCCTCTACGCCACCTTTTTTCCGATGG  1900

1901  CCCACAACACCGCCTCAACGCTTGAAGCCATGCTTAGAAACGACACCAAC  1950
      ||||||||||||||||||||||||||||||||||||||||||||||||||
1901  CCCACAACACCGCCTCAACGCTTGAAGCCATGCTTAGAAACGACACCAAC  1950

1951  GACCAGTCCTTTAACGACTACCTATCCGCCGCCAACATGCTTTACCCCAT  2000
      ||||||||||||||||||||||||||||||||||||||||||||||||||
1951  GACCAGTCCTTTAACGACTACCTATCCGCCGCCAACATGCTTTACCCCAT  2000

2001  ACCCGCCAACGCCACCAACGTGCCCATCTCTATCCCCTCGCGCAACTGGG  2050
      ||||||||||||||||||||||||||||||||||||||||||||||||||
2001  ACCCGCCAACGCCACCAACGTGCCCATCTCTATCCCCTCGCGCAACTGGG  2050
```

```
2051  CGGCTTTCCGAGGCTGGGCCTTTACGCGCCTTAAGACTAAGGAAACCCCA  2100
      ||||||||||||||||||||||||||||||||||||||||||||||||||
2051  CGGCTTTCCGAGGCTGGGCCTTTACGCGCCTTAAGACTAAGGAAACCCCA  2100

2101  TCCCTGGGTTCCGGCTACGACCCTTACTATACCTACTCTGGCTCCATACC  2150
      ||||||||||||||||||||||||||||||||||||||||||||||||||
2101  TCCCTGGGTTCCGGCTACGACCCTTACTATACCTACTCTGGCTCCATACC  2150

2151  CTACCTAGACGGAACCTTTTACCTTAATCACACCTTCAAAAAGGTGGCCA  2200
      ||||||||||||||||||||||||||||||||||||||||||||| ||||
2151  CTACCTAGACGGAACCTTTTACCTTAATCACACCTTCAAAAAGGTAGCCA  2200

2201  TCACCTTTGACTCTTCTGTTAGCTGGCCTGGCAATGACCGTCTGCTTACC  2250
      ||||||||||||||||||||||||||||||||||||||||||||||||||
2201  TCACCTTTGACTCTTCTGTTAGCTGGCCTGGCAATGACCGTCTGCTTACC  2250

2251  CCCAACGAGTTTGAGATCAAGCGTTCAGTTGACGGAGAGGGCTACAACGT  2300
      ||||||||||||||||||||||||||||||||||||||||||||||||||
2251  CCCAACGAGTTTGAGATCAAGCGTTCAGTTGACGGAGAGGGCTACAACGT  2300

2301  TGCCCAATGCAACATGACCAAAGACTGGTTCTTGGTACAGATGCTAGCCA  2350
      ||||||||||||||||||||||||||||||||||||||||||||||||||
2301  TGCCCAATGCAACATGACCAAAGACTGGTTCTTGGTACAGATGCTAGCCA  2350

2351  ACTACAACATAGGCTACCAGGGCTTTTATATCCCAGAAAGCTATAAGGAC  2400
      ||||||||||||||||||||||||||||||||||||||||||||||||||
2351  ACTACAACATAGGCTACCAGGGCTTTTATATCCCAGAAAGCTATAAGGAC  2400

2401  CGCATGTACTCCTTCTTTAGAAACTTCCAGCCCATGAGCCGTCAGGTGGT  2450
      ||||||||||||||||||||||||||||||||||||||||||||||||||
2401  CGCATGTACTCCTTCTTTAGAAACTTCCAGCCCATGAGCCGTCAGGTGGT  2450

2451  GGACGATACCAAATACAAGGACTACCAACAGGTGGGCATCCTCCACCAGC  2500
      ||||||||||||||||||||||||||||||||||||||||||||||||||
2451  GGACGATACCAAATACAAGGACTACCAACAGGTGGGCATCCTCCACCAGC  2500

2501  ACAATAACTCTGGCTTTGTTGGTTACCTCGCTCCCACCATGCGAGAGGGA  2550
      ||||||||||||||||||||||||||||||||||||||||||||||||||
2501  ACAATAACTCTGGCTTTGTTGGTTACCTCGCTCCCACCATGCGAGAGGGA  2550

2551  CAGGCCTACCCCGCCAACTTCCCCTACCCGCTTATAGGCAAGACCGCGGT  2600
      ||||||||||||||||||||||||||||||||||||||||||||||||||
2551  CAGGCCTACCCCGCCAACTTCCCCTACCCGCTTATAGGCAAGACCGCGGT  2600

2601  TGACAGTATTACCCAGAAAAAGTTTCTTTGCGACCGCACCCTTTGGCGCA  2650
      ||||||||||||||||||||||||||||||||||||||||||||||||||
2601  TGACAGTATTACCCAGAAAAAGTTTCTTTGCGACCGCACCCTTTGGCGCA  2650

2651  TTCCATTCTCCAGTAACTTTATGTCCATGGGTGCACTCACAGACCTGGGC  2700
      ||||||||||||||||||||||||||||||||||||||||||||||||||
2651  TTCCATTCTCCAGTAACTTTATGTCCATGGGTGCACTCACAGACCTGGGC  2700

2701  CAAAACCTTCTCTATGCAAACTCCGCCCACGCGCTAGATATGACTTTTGA  2750
      ||||||||||||||||||||||||||||||||||||||||||||||||||
2701  CAAAACCTTCTCTATGCAAACTCCGCCCACGCGCTAGATATGACTTTTGA  2750
```

```
2751  GGTGGATCCCATGGACGAGCCCACCCTTCTTTATGTTTTGTTTGAAGTCT    2800
      ||||||||||||||||||||||||||||||||||||||||||||||||||
2751  GGTGGATCCCATGGACGAGCCCACCCTTCTTTATGTTTTGTTTGAAGTCT    2800

2801  TTGACGTGGTCCGTGTGCACCAGCCGCACCGCGGCGTCATCGAGACCGTG    2850
      ||||||||||||||||||||||||||||||||||||||||||||||||||
2801  TTGACGTGGTCCGTGTGCACCAGCCGCACCGCGGCGTCATCGAGACCGTG    2850

2851  TACCTGCGCACGCCCTTCTCGGCCGGCAACGCCACAACAA..........    2900
      ||||||||||||||||||||||||||||||||||||||||
2851  TACCTGCGCACGCCCTTCTCGGCCGGCAACGCCACAACAA..........    2900
```

Mismatched sequences are printed in bold. The r-strands are shown.
