# Supplementary material for: Adenovirus Isolated From a Cat Is Related to Human Adenovirus 1
Source: Front Microbiol. 2019 Jun 25;10:1430. doi: 10.3389/fmicb.2019.01430 (PMC6603132; doi:10.3389/fmicb.2019.01430)
Supplement: Supplementary file 2 [file Data_Sheet_2.PDF]

Supplementary Figure 2. Comparison of the predicted amino acid sequence of the complete hexon polypeptide of HAdV-1 (D11) and FeAdV.

```
HAdV-1    1   ATPSMMPQWSYMHISGQDASEYLSPGLVQFARATETYFSLNNKFRNPTVA     50
              ||||||||||||||||||||||||||||||||||||||||||||||||||
FeAdV     1   ATPSMMPQWSYMHISGQDASEYLSPGLVQFARATETYFSLNNKFRNPTVA     50

         51   PTHDVTTDRSQRLTLRFIPVDREDTAYSYKARFTLAVGDNRVLDMASTYF    100
              ||||||||||||||||||||||||||||||||||||||||||||||||||
         51   PTHDVTTDRSQRLTLRFIPVDREDTAYSYKARFTLAVGDNRVLDMASTYF    100

        101   DIRGVLDRGPTFKPYSGTAYNALAPKGAPNSCEWEQEEPTQEMAEELEDE    150
              ||||||||||||||||||||||||||||||||||||||||||||||||||
        101   DIRGVLDRGPTFKPYSGTAYNALAPKGAPNSCEWEQEEPTQEMAEELEDE    150

        151   EEAEEEEAEEEAEAPQADQKVKKTHVYAQAPLAGEKITANGLQIVSDTQT    200
              ||||||||||||||||||||||||||||||||||||||||||||||||||
        151   EEAEEEEAEEEAEAPQADQKVKKTHVYAQAPLAGEKITANGLQIVSDTQT    200

        201   EGNPVFADPTYQPEPQVGESQWNEAEATASGGRVLKKTTPMKPCYGSYAR    250
              ||||||||||||||||||||||||||||||||||||||||||||||||||
        201   EGNPVFADPTYQPEPQVGESQWNEAEATASGGRVLKKTTPMKPCYGSYAR    250

        251   PTNKNGGQGILVANNQGALESKVEMQFFAPSGTAMNERNAVQPSIVLYSE    300
              ||||||||||||||||||||||||||||||||||||||||||||||||||
        251   PTNKNGGQGILVANNQGALESKVEMQFFAPSGTAMNERNAVQPSIVLYSE    300

        301   DVNMETPDTHISYKPSKTDENSKAMLGQQAMPNRPNYIAFRDNFIGLMYY    350
              ||||||||||||||||||||||||||||||||||||||||||||||||||
        301   DVNMETPDTHISYKPSKTDENSKAMLGQQAMPNRPNYIAFRDNFIGLMYY    350

        351   NSTGNMGVLAGQASQLNAVVDLQDRNTELSYQLLLDSIGDRTRYFSMWNQ    400
              ||||||||||||||||||||||||||||||||||||||||||||||||||
        351   NSTGNMGVLAGQASQLNAVVDLQDRNTELSYQLLLDSIGDRTRYFSMWNQ    400

        401   AVDSYDPDVRIIENHGTEDELPNYCFPLGGIGVTDTYQGIKSNGNGNPQN    450
              ||||||||||||||||||||||||||||||||||||||||||||||||||
        401   AVDSYDPDVRIIENHGTEDELPNYCFPLGGIGVTDTYQGIKSNGNGNPQN    450

        451   WTKNDDFAARNEIGVGNNFALEINLNANLWRNFLYSNIALYLPDKLKYTP    500
              ||||||||||||||||||||||||||||||||||||||||||||||||||
        451   WTKNDDFAARNEIGVGNNFALEINLNANLWRNFLYSNIALYLPDKLKYTP    500

        501   TNVEISPNPNSYDYMNKRVVAPGLVDCYINLGARWSLDYMDNVNPFNHHR    550
              ||||||||||||||||||||||||||||||||||||||||||||||||||
        501   TNVEISPNPNSYDYMNKRVVAPGLVDCYINLGARWSLDYMDNVNPFNHHR    550

        551   NAGLRYRSMLLGNGRYVPFHIQVPQKFFAIKNLLLLPGSYTYEWNFRKDV    600
              ||||||||||||||||||||||||||||||||||||||||||||||||||
        551   NAGLRYRSMLLGNGRYVPFHIQVPQKFFAIKNLLLLPGSYTYEWNFRKDV    600

        601   NMVLQSSLGNDLRVDGASIKFDSICLYATFFPMAHNTASTLEAMLRNDTN    650
              ||||||||||||||||||||||||||||||||||||||||||||||||||
        601   NMVLQSSLGNDLRVDGASIKFDSICLYATFFPMAHNTASTLEAMLRNDTN    650
```

```
651  DQSFNDYLSAANMLYPIPANATNVPISIPSRNWAAFRGWAFTRLKTKETP   700
     ||||||||||||||||||||||||||||||||||||||||||||||||||
651  DQSFNDYLSAANMLYPIPANATNVPISIPSRNWAAFRGWAFTRLKTKETP   700

701  SLGSGYDPYYTYSGSIPYLDGTFYLNHTFKKVAITFDSSVSWPGNDRLLT   750
     ||||||||||||||||||||||||||||||||||||||||||||||||||
701  SLGSGYDPYYTYSGSIPYLDGTFYLNHTFKKVAITFDSSVSWPGNDRLLT   750

751  PNEFEIKRSVDGEGYNVAQCNMTKDWFLVQMLANYNIGYQGFYIPESYKD   800
     ||||||||||||||||||||||||||||||||||||||||||||||||||
751  PNEFEIKRSVDGEGYNVAQCNMTKDWFLVQMLANYNIGYQGFYIPESYKD   800

801  RMYSFFRNFQPMSRQVVDDTKYKDYQQVGILHQHNNSGFVGYLAPTMREG   850
     ||||||||||||||||||||||||||||||||||||||||||||||||||
801  RMYSFFRNFQPMSRQVVDDTKYKDYQQVGILHQHNNSGFVGYLAPTMREG   850

851  QAYPANFPYPLIGKTAVDSITQKKFLCDRTLWRIPFSSNFMSMGALTDLG   900
     ||||||||||||||||||||||||||||||||||||||||||||||||||
851  QAYPANFPYPLIGKTAVDSITQKKFLCDRTLWRIPFSSNFMSMGALTDLG   900

901  QNLLYANSAHALDMTFEVDPMDEPTLLYVLFEVFDVVRVHQPHRGVIETV   950
     ||||||||||||||||||||||||||||||||||||||||||||||||||
901  QNLLYANSAHALDMTFEVDPMDEPTLLYVLFEVFDVVRVHQPHRGVIETV   950

951  YLRTPFSAGNATT.....................................  1000
     |||||||||||||
951  YLRTPFSAGNATT.....................................  1000
```

One letter symbols for the amino acids are: A=alanine, C=cysteine, D=aspartic acid, E=glutamic acid, F=phenyl-alanine, G=glycine, H=histidine, I=isoleucine, K=lysine, L=leucine, M=methionine, N=asparagine, P=proline, Q=glutamine, R=arginine, S=serine, T=threonine, V=valine, W=tryptophan, Y=tyrosine.
