# Supplementary material for: Adenovirus Isolated From a Cat Is Related to Human Adenovirus 1
Source: Front Microbiol. 2019 Jun 25;10:1430. doi: 10.3389/fmicb.2019.01430 (PMC6603132; doi:10.3389/fmicb.2019.01430)
Supplement: Supplementary file 3 [file Data_Sheet_3.PDF]

Supplementary Figure 3. Sequence alignment of the complete fiber gene of HAdV-1 (D11) and FeAdV.

```
HAdV-1    1  ATGAAGCGCGCCAGACCGTCTGAAGACACCTTCAACCCCGTGTACCCTTA     50
             ||||||||||||||||||||||||||||||||||||||||||||||||||
FeAdV     1  ATGAAGCGCGCCAGACCGTCTGAAGACACCTTCAACCCCGTGTACCCTTA     50

         51  TGACACGGAAACCGGCCCCCAACAGTGCCTTTCCTTACCCCTCCCTTTG    100
             ||||||||||||||||||||||||||||||||||||||||||||||||||
         51  TGACACGGAAACCGGCCCCCAACAGTGCCTTTCCTTACCCCTCCCTTTG    100

        101  TCTCCCCCAATGGGTTCCAAGAAAGTCCCCCTGGGGTGCTTTCTCTGCGC    150
             ||||||||||||||||||||||||||||||||||||||||||||||||||
        101  TCTCCCCCAATGGGTTCCAAGAAAGTCCCCCTGGGGTGCTTTCTCTGCGC    150

        151  CTTTCCGAACCACTCGTCACCTCCCACGGCATGCTTGCGCTCAAAATGGG    200
             ||||||||||||||||||||||||||||||||||||||||||||||||||
        151  CTTTCCGAACCACTCGTCACCTCCCACGGCATGCTTGCGCTCAAAATGGG    200

        201  CAGCGGCCTAGCCCTGGACGAGGCCGGCAACCTTACATCACAAAATATAA    250
             ||||||||||||||||||| ||||||||||||||||||||||||||||||
        201  CAGCGGCCTAGCCCTGGACAAGGCCGGCAACCTTACATCACAAAATATAA    250

        251  CCACCGTCACTGAGCCATTAAAAAAAACAAAGTCCAACATAAGCCTGGAA    300
             ||||||||||||||||||||||||||||||||||||||||||||||||||
        251  CCACCGTCACTGAGCCATTAAAAAAAACAAAGTCCAACATAAGCCTGGAA    300

        301  ACCTCCGCGCCCCTCACAGTTACATCAGGCATTCTTACCGTAGCCACTGC    350
             ||||||||||||||||||||||||||||||||||||||||||||||||||
        301  ACCTCCGCGCCCCTCACAGTTACATCAGGCATTCTTACCGTAGCCACTGC    350

        351  CGCACCTTTGGTGGTCGCGGGCAACAGTCTTACCGTGCAGTCACAGGCCC    400
             ||||||||||||||||||||||||||||||||||||||||||||||||||
        351  CGCACCTTTGGTGGTCGCGGGCAACAGTCTTACCGTGCAGTCACAGGCCC    400

        401  CGTTAACCGTGCAAGACTCTAAACTTAGCATTGCCACCAAAGGACCCCTC    450
             ||||||||||||||||||||||||||||||||||||||||||||||||||
        401  CGTTAACCGTGCAAGACTCTAAACTTAGCATTGCCACCAAAGGACCCCTC    450

        451  ACAGTGTCAGAAGGAAAGCTGGCCCTGCAAACATCGGGCCCCCTTTCTGC    500
             ||||||||||||||||||||||||||||||||||||||||||||||||||
        451  ACAGTGTCAGAAGGAAAGCTGGCCCTGCAAACATCGGGCCCCCTTTCTGC    500

        501  CACTGATAACAACACCCTTACTATTACTACTTCACCCCCTATAACTACTA    550
             ||||||||||||||||||||||||||||||||||||||||||||||||||
        501  CACTGATAACAACACCCTTACTATTACTACTTCACCCCCTATAACTACTA    550

        551  CAAATGGTAGTTTGGGTGTTAACATGGAAAACCCACTTTACAACAGCAAT    600
             |||||||||||||||||||||||||||||||||||||||||||||| ||||
        551  CAAATGGTAGTTTGGGTGTTAACATGGAAAACCCACTTTACAACAACAAT    600

        601  GGCAAACTGGGGCTCAGAGTTGCAGGTCCTTTGCAAGTGACCAACGACTC    650
             ||||||||||||||||||||||||||||||||||||||||||||||||||
        601  GGCAAACTGGGGCTCAGAGTTGCAGGTCCTTTGCAAGTGACCAACGACTC    650
```

```
651   TCACGCACTAACAGTAGGTACAGGTCAGGGTGTTGCCATTGATAATAATG   700
      ||||||||||||||||||||||||||||||||||||||||||||||||||
651   TCACGCACTAACAGTAGGTACAGGTCAGGGTGTTGCCATTGATAATAATG   700

701   CGCTACACACAAAAGTTACAGGCGCAATAGGGTATGATACATCTGGTAAC   750
      ||||||||||||||||||||||||||||||||||||||||||||||||||
701   CGCTACACACAAAAGTTACAGGCGCAATAGGGTATGATACATCTGGTAAC   750

751   ATGGAACTTAAAACTGGAGGGGGAGTGCGTGTAGACAGTGTAAATAGGCG   800
      ||||||||||||||||||||||||||||||||||||||||||||||||||
751   ATGGAACTTAAAACTGGAGGGGGAGTGCGTGTAGACAGTGTAAATAGGCG   800

801   TCTTATACTCGATGTTGATTATCCATTTGATGCCCAAAGCCAACTACGCC   850
      ||||||||||||||||||||||||||||||||||||||||||||||||||
801   TCTTATACTCGATGTTGATTATCCATTTGATGCCCAAAGCCAACTACGCC   850

851   TAAAACTAGGACAGGGACCCCTATATGTAAACTCAAGCACACACAACTTG   900
      ||||||||||||||||||||||||||||||||||||||||||||||||||
851   TAAAACTAGGACAGGGACCCCTATATGTAAACTCAAGCACACACAACTTG   900

901   GATTTAAACTACAATAAAGGCCTTCACTTGTTTACAACTGGAAACTCTAA   950
      ||||||||||||||||||||||||||||||||||||||||||||||||||
901   GATTTAAACTACAATAAAGGCCTTCACTTGTTTACAACTGGAAACTCTAA   950

951   AAAGCTAGAAGTAAATCTTAAAACAACAAAAGGTCTTATTTTTGACACCG   1000
      ||||||||||||||||||||||||||||||||||||||||||||||||||
951   AAAGCTAGAAGTAAATCTTAAAACAACAAAAGGTCTTATTTTTGACACCG   1000

1001  ATGCTGTTGCAATAAATGCAGCACAAGGTCTAGAATTTGGTAATGATACG   1050
      |||||||||||||| |||||||||||||||||||||||||||||||||||
1001  ATGCTGTTGCAATACATGCAGCACAAGGTCTAGAATTTGGTAATGATACG   1050

1051  TCAACAAATACAAACCCACTTAAAACAAAACTAGGATTGGGATTGGACTA   1100
      ||||||||||||||||||||||||||||||||||||||||||||||||||
1051  TCAACAAATACAAACCCACTTAAAACAAAACTAGGATTGGGATTGGACTA   1100

1101  TGACTCAAACGGTGGTATGATTCCCAAACTTGGAACAGGCCTTAGTTTTG   1150
      ||||||||||||||||||||||||||||||||||||||||||||||||||
1101  TGACTCAAACGGTGGTATGATTCCCAAACTTGGAACAGGCCTTAGTTTTG   1150

1151  ATACTACAGGGGCTATAACAGTGGGAAACAAAAGCGATGACAAACTTACC   1200
      ||||||||||||||||||||||||||||||||||||||||||||||||||
1151  ATACTACAGGGGCTATAACAGTGGGAAACAAAAGCGATGACAAACTTACC   1200

1201  CTGTGGACAACTCCTGACCCATCACCTAATTGTCAAATATACTCAGAAAA   1250
      ||||||||||||||||||||||||||||||||||||||| ||||||||||
1201  CTGTGGACAACTCCTGACCCATCACCTAATTGTCAAATACACTCAGAAAA   1250

1251  AGATGCTAAGCTAACACTAGTTTTAACTAAATGTGGCAGTCAGGTACTTG   1300
      ||||||||||||||||||||||||||||||||| ||||||||||||||||
1251  AGATGCTAAGCTAACACTAGTTTTAACTAAATGCGGCAGTCAGGTACTTG   1300

1301  CAACTGTGTCAGCACTGGCTGTAAAGGGCAGCCTAGCTCCCATTAGCGGA   1350
      |||||||||||||||||||||||| ||||||||||||||| |||||||||
1301  CAACTGTGTCAGCACTGGCTGTAAGGGGCAGCCTAGCTCCTATTAGCGGA   1350
```

1351 ACAATAAGTAGTGCTCACATTATTCTCAGATTTAATGAACATGG**G**GTGCT 1400

|||||||||||||||||||||||||||||||||||||||||||| |||||

1351 ACAATAAGTAGTGCTCACATTATTCTCAGATTTAATGAACATGG**A**GTGCT 1400

1401 AATGAATCATTCT**G**GTTTGGATCCCCAATACTGGAATTTCAGAAAAGGGG 1450

||||||||||||| ||||||||||||||||||||||||||||||||||||

1401 AATGAATCATTCT**A**GTTTGGATCCCCAATACTGGAATTTCAGAAAAGGGG 1450

1451 ATTTAACAAACGCTACAGCATATACTAACGCAGTTGGTTTTATGCCCAAC 1500

||||||||||||||||||||||||||||||||||||||||||||||||||

1451 ATTTAACAAACGCTACAGCATATACTAACGCAGTTGGTTTTATGCCCAAC 1500

1501 CTTAAAGCTTACCCAAAAACTCAAAGTAGAACTGCAAAAAGCAACATTGT 1550

||||||||||||||||||||||||||||||||||||||||||||||||||

1501 CTTAAAGCTTACCCAAAAACTCAAAGTAGAACTGCAAAAAGCAACATTGT 1550

1551 TAGTCAGGTTTATCTTAATGGAGAAAAAGA**T**AAACCAATGACACTCACTA 1600

|||||||||||||||||||||||||||||| |||||||||||||||||||

1551 TAGTCAGGTTTATCTTAATGGAGAAAAAGA**A**AAACCAATGACACTCACTA 1600

1601 TTACACTTAATGGAACTGATGAAAATCAAACCACTCCCGCCAGTACATAC 1650

||||||||||||||||||||||||||||||||||||||||||||||||||

1601 TTACACTTAATGGAACTGATGAAAATCAAACCACTCCCGCCAGTACATAC 1650

1651 TCAATTTC**A**TTTTCATGGAGCTGGCCTAGCAATCAAACATACATTGGTCA 1700

|||||||| |||||||||||||||||||||||||||||||||||||||||

1651 TCAATTTC**C**TTTTCATGGAGCTGGCCTAGCAATCAAACATACATTGGTCA 1700

1701 AACATTTGCCACTAATTCCTACACCTTCTCCTACATTGCCCA**A**GAATAA. 1750

|||||||||||||||||||||||||||||||||||||||||| ||||||

1701 AACATTTGCCACTAATTCCTACACCTTCTCCTACATTGCCCA**G**GAATAA. 1750

Mismatched sequences are printed in bold. The r-strands are shown.
