# Supplementary material for: Adenovirus Isolated From a Cat Is Related to Human Adenovirus 1
Source: Front Microbiol. 2019 Jun 25;10:1430. doi: 10.3389/fmicb.2019.01430 (PMC6603132; doi:10.3389/fmicb.2019.01430)
Supplement: Supplementary file 4 [file Data_Sheet_4.PDF]

Supplementary Figure 4. Comparison of the predicted amino acid sequence of the complete fiber of HAdV-1 (D11) and FeAdV.

```
            ↓Tail                                       ↓Shaft
HAdV-1   1  MKRARPSEDTFNPVYPYDTETGPPTVPFLTPPFVSPNGFQESPPGVLSLR       50
            ||||||||||||||||||||||||||||||||||||||||||||||||||
FeAdV    1  MKRARPSEDTFNPVYPYDTETGPPTVPFLTPPFVSPNGFQESPPGVLSLR       50
                     →2             →3                 →4
        51  LSEPLVTSHGMLALKMGSGLALDEAGNLTSQNITTVTEPLKKTKSNISLE      100
            |||||||||||||||||||||||| |||||||||||||||||||||||||
        51  LSEPLVTSHGMLALKMGSGLALDKAGNLTSQNITTVTEPLKKTKSNISLE      100
                  →5             →6             →7
       101  TSAPLTVTSGILTVATAAPLVVAGNSLTVQSQAPLTVQDSKLSIATKGPL      150
            ||||||||||||||||||||||||||||||||||||||||||||||||||
       101  TSAPLTVTSGILTVATAAPLVVAGNSLTVQSQAPLTVQDSKLSIATKGPL      150
                      →8               →9                    →10
       151  TVSEGKLALQTSGPLSATDNNTLTITTSPPITTTNGSLGVNMENPLYNSN      200
            |||||||||||||||||||||||||||||||||||||||||||||||| |
       151  TVSEGKLALQTSGPLSATDNNTLTITTSPPITTTNGSLGVNMENPLYNNN      200
                           →11              →12              →13
       201  GKLGLRVAGPLQVTNDSHALTVGTGQGVAIDNNALHTKVTGAIGYDTSGN      250
            ||||||||||||||||||||||||||||||||||||||||||||||||||
       201  GKLGLRVAGPLQVTNDSHALTVGTGQGVAIDNNALHTKVTGAIGYDTSGN      250
                        →14                  →15          →16
       251  MELKTGGGVRVDSVNRRLILDVDYPFDAQSQLRLKLGQGPLYVNSSTHNL      300
            ||||||||||||||||||||||||||||||||||||||||||||||||||
       251  MELKTGGGVRVDSVNRRLILDVDYPFDAQSQLRLKLGQGPLYVNSSTHNL      300
                            →17              →18            →19
       301  DLNYNKGLHLFTTGNSKKLEVNLKTTKGLIFDTDAVAINAAQGLEFGNDT      350
            |||||||||||||||||||||||||||||||||||||| |||||||||||
       301  DLNYNKGLHLFTTGNSKKLEVNLKTTKGLIFDTDAVAIHAAQGLEFGNDT      350
                     →20        →21          →22
       351  STNTNPLKTKLGLGLDYDSNGGMIPKLGTGLSFDTTGAITVGNKSDDKLT      400
            ||||||||||||||||||||||||||||||||||||||||||||||||||
       351  STNTNPLKTKLGLGLDYDSNGGMIPKLGTGLSFDTTGAITVGNKSDDKLT      400
              ↓Knob          →B       ←  →C        ←
       401  LWTTPDPSPNCQIYSEKDAKLTLVLTKCGSQVLATVSALAVKGSLAPISG      450
            ||||||||||||| ||||||||||||||||||||||||||| ||||||||
       401  LWTTPDPSPNCQIHSEKDAKLTLVLTKCGSQVLATVSALAVRGSLAPISG      450
               →D     ←                 →E ←  →←F
       451  TISSAHIILRFNEHGVLMNHSGLDPQYWNFRKGDLTNATAYTNAVGFMPN      500
            ||||||||||||||||||||| ||||||||||||||||||||||||||||
       451  TISSAHIILRFNEHGVLMNHSSLDPQYWNFRKGDLTNATAYTNAVGFMPN      500
                          →G    ←      →H     ←
       501  LKAYPKTQSRTAKSNIVSQVYLNGEKDKPMTLTITLNGTDENQTTPASTY      550
            |||||||||||||||||||||||||| |||||||||||||||||||||||
       501  LKAYPKTQSRTAKSNIVSQVYLNGEKEKPMTLTITLNGTDENQTTPASTY      550
                   →I     ←        →J  ←
       551  SISFSWSWPSNQTYIGQTFATNSYTFSYIAQE*.................      600
            |||||||||||||||||||||||||||||||||
       551  SISFSWSWPSNQTYIGQTFATNSYTFSYIAQE*.................      600
```

The tail, shaft and knob regions are marked by vertical arrows. Mismatched amino acids are printed in bold. In the shaft region, horizontal arrows show the beginning of pseudorepeat motifs. In the tail region, right and left pointing horizontal arrows define β-strands A to J. The unique RGS motif is

printed in bold italic. One letter symbols for the amino acids are: A=alanine, C=cysteine, D=aspartic acid, E=glutamic acid, F=phenyl-alanine, G=glycine, H=histidine, I=isoleucine, K=lysine, L=leucine, M=methionine, N=asparagine, P=proline, Q=glutamine, R=arginine, S=serine, T=threonine, V=valine, W=tryptophan, Y=tyrosine.
